# Editorial: Host response to veterinary infectious diseases: role of coding and non-coding RNAs as biomarkers and disease modulators

**DOI:** 10.3389/fvets.2023.1275169

**Published:** 2023-08-29

**Authors:** Mohamed Samir

**Affiliations:** ^1^The Immunogenetics Group, The Pirbright Institute, Woking, United Kingdom; ^2^Department of Zoonoses, Faculty of Veterinary Medicine, Zagazig University, Zagazig, Egypt

**Keywords:** miRNA, piRNA, long non-coding, infectious diseases, veterinary medicine

## Introduction

The motivation behind hosting and maintaining this Research Topic is the interest in the roles of host non-coding RNAs in infectious and zoonotic diseases, which we believe is underappreciated in veterinary medicine, at least relatively to its counterpart in humans. The latter is fueled by the evolving role of these molecules in human cancer research (1). The aim of this topic is not only to draw scientific attention to the involvement of coding RNAs (i.e., mRNAs) in veterinary infectious diseases but also to portray their intricate relations with non-coding RNAs (sncRNAs), which feature multiple species such as microRNAs (miRNAs), small nuclear RNAs (snRNAs), small nucleolar RNAs (snoRNAs), and Piwi-interacting RNAs (piRNAs), in addition to long non-coding RNAs (lncRNAs). Aspects such as their regulation in animal infection with various pathogens, their biomarker potential, and their biogenesis were and will continue to be covered here. After almost 2 years of publishing this topic within the remit of *Frontiers in Veterinary Sciences*, we are continuing at a steady and successful pace to attain our anticipated impact. With six articles being published, the topic has witnessed a big leap in article views, from 2,354 views at its launch in 2021 to around 13,000 views in July 2023. These numbers, along with other statistics shown on the topic website, already mirror the uniqueness of this field and calls for further in-depth exploration.

## RNAs in veterinary infectious diseases: where we are: reflections from published articles in the topic

**Figure 1:**
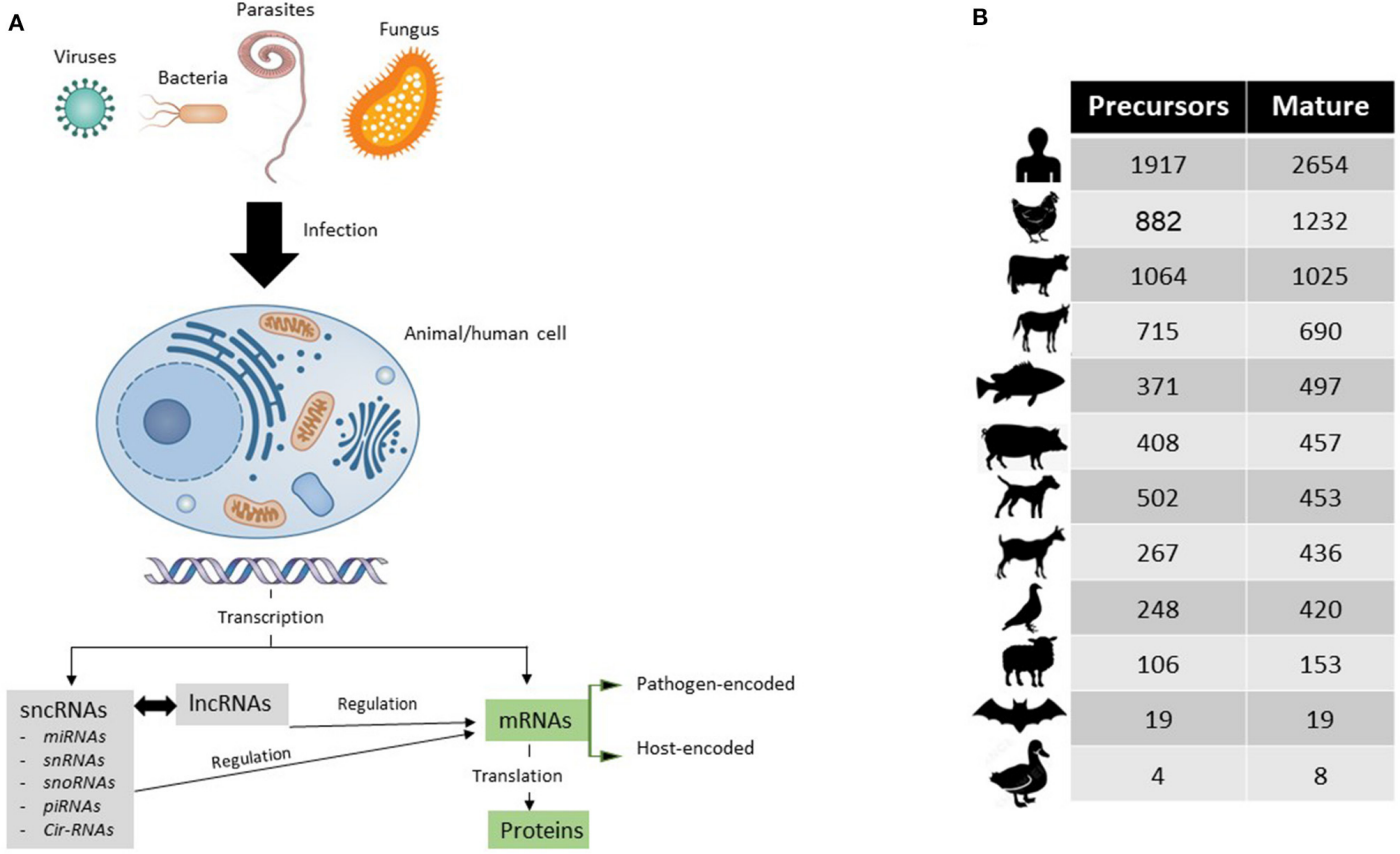
**(A)** Simplified schematic representation of how coding (mRNAs) and non-coding RNAs (sncRNAs and lncRNAs) are triggered and act during infection of human or animal cell with a pathogen of interest. **(B)** Up-to-date numbers of annotated miRNAs in their precursors (immature) and mature (functional) forms in humans and other animal species of veterinary importance.

Around 80% of the genomic DNA is transcribed, leaving out 20% as the non-transcribed portion, the so-called junk DNA (2). Only 1–2% of the transcribed mRNAs are translated to protein, and the remaining 78% encompasses ncRNAs that can be either small sncRNAs (< 200 nts) or long lncRNAs (>200 nts) (3). These ncRNAs regulate the functionality of other mRNAs (Figure 1A).

Studies on mRNAs have been exploring wide aspects of the pathobiology of significant viruses such as avian influenza viruses in chickens (4, 5) and bovine viral diarrhea in bovines (6), zoonotic parasites such as toxoplasma (7) and cryptosporidium (8), and economically important pathogens that cause mastitis in bovines or caprine (9, 10). In virtue of small RNA sequencing, similar studies are being conducted at a limited scale on miRNAs and other sncRNAs. Both sncRNAs and lncRNAs could fine tune gene expression at the transcriptional stage and posttranscription (11). Since the discovery of the first microRNAs (miRNAs), named as *lin4*, in 1993 by Lee et al. in *C. elegans* (12), an obvious growth of miRNA numbers has been evidenced. By now, 38,589 miRNAs have been annotated in the most recent version of miRbase (release 22.1) (13). Figure 1B shows the most recent numbers of annotated miRNAs in the miRbase database.

It is not surprising that mastitis, a tricky problem in dairy farming that causes decline of milk quality and remarkable economic losses (14), has become an active area of research for sncRNAs. The signature and potential function of lncRNA associated with *Staphylococcus aureus*-caused mastitis in bovines have been explored in some studies (15). Comparable efforts were conducted for miRNAs in the same context (16, 17). An article in our Research Topic (Luoreng et al.) tried to tackle this problem and highlighted the roles of upregulated miRNAs (miR-320a, miR-19a, and miR-19b) and other downregulated ones (miR-143, miR-205, and miR-24) as diagnostic markers for *Staphylococcus aureus*-caused mastitis at the 5^th^ and 7^th^ day in an experimentally infected animals. MiRNA regulatory functions have been also revealed in a plethora of infectious diseases affecting animals [comprehensive reviews are available here (18–20)].

Given the epigenomic regulatory role of sncRNAs on mRNA genes, a common approach is to dually investigate the changes in sncRNAs and their target mRNAs. This would provide a holistic picture of what is operating during an infection event (21). A study published in our topic by Chen Q. et al. exemplifies this trend in canine distemper virus infection in an understudied host, the mink. The authors found that mir-140-5p and mir-378-12 targeted corresponding mRNA genes in the NF-kappa B signaling pathway. The JAK-STAT signaling pathway was found to be regulated by mir-425-2, mir-139-4, mir-140-6, mir-145-3, mir-140-5p, and mir-204-2. Similar studies were also performed in the context of avian influenza infection, yet these focused on mRNA-lncRNAs interaction (22). Another research line has been the RNA interference (RNAi), a phenomenon that involves an interaction between natural or artificially introduced RNAs into the cell to physically bind a target mRNA, thus leading to gene silencing (23). The role of RNAi has just emerged in the veterinary infectious disease field. A plethora of studies have studied the consequence of this process on viruses (24–26) and parasites of veterinary importance (27, 28). As shown in one of the articles published in this topic, short hairpin RNA (shRNA), a form of RNAi, was found to suppress the replication of border disease virus (Hajihasani Arani et al.). Border disease is a viral sickness of small ruminants and pigs (29) that might lead to symptoms such as infertility, abortion, stillbirth, and the birth of tiny, faint young (30).

## Knowledge gap: aspects to be addressed in future research

Highlighted here are some knowledge gaps that still need to be addressed. With the expanding universe of sncRNAs (31), some of their species have remained superficially studied or have not been investigated at all in the context of animal infectious diseases. PiRNAs, snoRNAs, and circular RNAs (circRNAs) are examples of these overlooked molecules. Using a duck model for H5N1 influenza virus, we have identified an organ-specific expression pattern of piRNAs (32). Others have investigated roles of piRNAs in insects that could allow to combat insect-borne diseases (33). Interestingly, the genes involved in the piRNA pathway are more rapidly evolving compared with other RNAi genes. Our Research Topic is becoming a platform to explore some of these overlooked molecules. The article published by Chen L. et al. showed that circular RNAs interacting with miRNAs and mRNAs could shape the host response to Newcastle disease virus (NDV), a devastating chicken virus. They found that many circular RNAs were differentially regulated and can determine the NDV-induced metabolic changes in chicken embryo fibroblast cells via regulating mRNA and miRNAs. They identified that just one of these circular RNAs, circ-EZH2, inhibited NDV replication when overexpressed, indicating that circRNAs are involved in NDV replication. These studies and others call for additional research on this topic. An annotation database also needs to be continuously built, refined, and further completed for ncRNAs in less annotated animal species. Surprisingly enough, miRbase, the miRNA database, lacks annotation for miRNAs in cats, an important feline species. A broad yet valid question is to what the extent the epigenomic and regulatory logic of these sncRNAs is conserved across animals and how this contributes to zoonotic potential or a cross-species transmission of the pathogen. It is very important for researchers in this field to remember that *in vitro* models of infection (e.g., cell lines and organ cultures) are by no means a reflection of the mechanisms occurring in the respective natural animal hosts of the pathogens of interest. Similarly, the use of non-traditional animal models poses challenges of background genetic differences, rendering the model non-representative. Considering the economic deficits in middle- and low-income countries, which cast a shadow on the lack of funds given to researchers, it is recommended to keep the studies as complete as possible, even if conducted on a small scale, in a way that enables reaching solid conclusions.

Altogether, the field of coding and non-coding RNAs is rapidly evolving, and the roles of these molecules in the pathogenesis and outcome of veterinary infectious diseases are being continuously discovered. This Research Topic represents a platform to explore these aspects. The investigation of these molecules at the host level could lead to remarkable discoveries, from biomarkers for infection to therapeutic avenues, that researchers and veterinarians could use for combating veterinary infectious diseases.

## Author contributions

MS: Conceptualization, Visualization, Writing—original draft.

## References

[1] López-Camarillo C Slaby O Silva-Cázares MB. Strategic molecular biomarkers and microRNAs in cancer. Front Oncol. (2022) 12:1031349. 10.3389/fonc.2022.1031349 36313649 PMC9598417

[2] Fagundes NJ Bisso-Machado R Figueiredo PI Varal M Zani AL. What we talk about when we talk about “junk DNA”. Genome Biol Evolut. (2022) 14:evac055. 10.1093/gbe/evac055 35535669 PMC9086759

[3] Mudge JM Harrow J. Creating reference gene annotation for the mouse C57BL6/J genome assembly. Mammalian Genome. (2015) 26:366–78. 10.1007/s00335-015-9583-x 26187010 PMC4602055

[4] Zhang W Li H Cheng G Hu S Li Z Bi D. Avian influenza virus infection induces differential expression of genes in chicken kidney. Res Vet Sci. (2008) 84:374–81. 10.1016/j.rvsc.2007.05.015 17692877

[5] Dar A Munir S Vishwanathan S Manuja A Griebel P Tikoo S. Transcriptional analysis of avian embryonic tissues following infection with avian infectious bronchitis virus. Virus Res. (2005) 110:41–55. 10.1016/j.virusres.2005.01.006 15845254 PMC7114260

[6] Liu C Liu Y Liang L Cui S Zhang Y. RNA-Seq based transcriptome analysis during bovine viral diarrhoea virus (BVDV) infection. BMC Genomics. (2019) 20:1–8. 10.1186/s12864-019-6120-4 31651237 PMC6813989

[7] Cui J Shen B. Transcriptomic analyses reveal distinct response of porcine macrophages to Toxoplasma gondii infection. Parasitol Res. (2020) 119:1819–28. 10.1007/s00436-020-06677-5 32399721

[8] Mirhashemi ME Noubary F Chapman-Bonofiglio S Tzipori S Huggins GS Widmer G. Transcriptome analysis of pig intestinal cell monolayers infected with Cryptosporidium parvum asexual stages. Parasit Vectors. (2018) 11:1–9. 10.1186/s13071-018-2754-3 29530089 PMC5848449

[9] Mitra SD Ganaie F Bankar K Velu D Mani B Vasudevan M. Genome-wide analysis of mammary gland shows modulation of transcriptome landscape with alternative splice variants in Staphylococcus aureus mastitis in mice. Gene. (2020) 735:144278. 10.1016/j.gene.2019.144278 31821873

[10] Xuan R Wang J Zhao X Li Q Wang Y Du S. Transcriptome analysis of goat mammary gland tissue reveals the adaptive strategies and molecular mechanisms of lactation and involution. Int J Mol Sci. (2022) 23:14424. 10.3390/ijms232214424 36430911 PMC9693614

[11] Lieberman J. Tapping the RNA world for therapeutics. Nat Struct Mol Biol. (2018) 25:357–64. 10.1038/s41594-018-0054-4 29662218 PMC6052442

[12] Lee RC Feinbaum RL Ambros V. The C. elegans heterochronic gene lin-4 encodes small RNAs with antisense complementarity to lin-14. Cell. (1993) 75:843–54. 10.1016/0092-8674(93)90529-Y 8252621

[13] Kozomara A Birgaoanu M Griffiths-Jones S. miRBase: from microRNA sequences to function. Nucleic Acids Res. (2019) 47:D155–62. 10.1093/nar/gky1141 30423142 PMC6323917

[14] Halasa T Huijps K Østerås O Hogeveen H. Economic effects of bovine mastitis and mastitis management: a review. Vet Quarterly. (2007) 29:18–31. 10.1080/01652176.2007.9695224 17471788

[15] Mi S Tang Y Dari G Shi Y Zhang J Zhang H. Transcriptome sequencing analysis for the identification of stable lncRNAs associated with bovine Staphylococcus aureus mastitis. J Anim Sci Biotechnol. (2021) 12:1–7. 10.1186/s40104-021-00639-2 34895356 PMC8667444

[16] Jin W Ibeagha-Awemu EM Liang G Beaudoin F Zhao X Guan LL. Transcriptome microRNA profiling of bovine mammary epithelial cells challenged with *Escherichia coli* or *Staphylococcus* aureusbacteria reveals pathogen directed microRNA expression profiles. BMC Genomics. (2014) 15:1–6. 10.1186/1471-2164-15-181 24606609 PMC4029070

[17] Pu J Li R Zhang C Chen D Liao X Zhu Y. Expression profiles of miRNAs from bovine mammary glands in response to Streptococcus agalactiae-induced mastitis. J Dairy Res. (2017) 84:300–8. 10.1017/S0022029917000437 28831974

[18] Do DN Dudemaine PL Mathur M Suravajhala P Zhao X Ibeagha-Awemu EM. regulatory functions in farm animal diseases, and biomarker potentials for effective therapies. Int J Mol Sci. (2021) 22:3080. 10.3390/ijms22063080 33802936 PMC8002598

[19] Samir M Vaas LA Pessler F. MicroRNAs in the host response to viral infections of veterinary importance. Front Vet Sci. (2016) 3:86. 10.3389/fvets.2016.00086 27800484 PMC5065965

[20] Samir M Pessler F. Small non-coding RNAs associated with viral infectious diseases of veterinary importance: potential clinical applications. Front Vet Sci. (2016) 3:22. 10.3389/fvets.2016.00022 27092305 PMC4819147

[21] Tucker AR Salazar NA Ayoola AO Memili E Thomas BN Morenikeji OB. Regulatory network of miRNA, lncRNA, transcription factor and target immune response genes in bovine mastitis. Sci Rep. (2021) 11:21899. 10.1038/s41598-021-01280-9 34753991 PMC8578396

[22] Wang Q Wang Z Zhang J Zhang Q Zheng M Wen J. Dual RNA-Seq of H5N1 avian influenza virus and host cell transcriptomes reveals novel insights into host-pathogen cross talk. Front Microbiol. (2022) 13:828277. 10.3389/fmicb.2022.828277 35495687 PMC9039741

[23] Hannon GJ RNA. interference. Nature. (2002) 418:244–51. 10.1038/418244a 12110901

[24] Wu HX Wang HL Guo XF Yang YJ Wang TC Gao YW. Adeno-associated viruses serotype 2-mediated rna interference efficiently inhibits rabies virus replication *in vitro* and *in vivo*. J Vet Med Sci. (2013) 75:1355–61. 10.1292/jvms.13-0127 23774028 PMC3942934

[25] Dietrich I Jansen S Fall G Lorenzen S Rudolf M Huber K. RNA interference restricts Rift Valley Fever virus in multiple insect systems. Msphere. (2017) 2:e00090–17. 10.1128/mSphere.00090-17 28497117 PMC5415632

[26] Villa R Renzi S Dotti S Lucchini F. siRNAs pools generated in Escherichia coli exhibit strong RNA-interference activity against influenza virus genomic sequences. Virology. (2023) 579:38–45. 10.1016/j.virol.2022.12.013 36599198

[27] Rinaldi G Dell'Oca N Castillo E Tort JF. Gene silencing in the liver fluke Fasciola hepatica: RNA interference Fasciola hepatica. Methods Prot. (2020) 3:67–92. 10.1007/978-1-0716-0475-5_6 32399922

[28] Holmes M Itaas V Ananvoranich S. Sustained translational repression of lactate dehydrogenase 1 in Toxoplasma gondii bradyzoites is conferred by a small regulatory RNA hairpin. FEBS J. (2014) 281:5077–91. 10.1111/febs.13048 25223457

[29] Dastjerdi A Strong R La Rocca SA Wessels M Wessels J Whitaker K. Investigation into an outbreak of Border disease virus in pigs in England. (2022) 69:1698–706. 10.1111/tbed.14539 35353447 PMC9544453

[30] Løken T. Border disease in sheep. Vet Clin North Am Food Animal Pract. (1995) 11:579–95. 10.1016/S0749-0720(15)30468-0 8581864

[31] Shi J Zhou T Chen Q. Exploring the expanding universe of small RNAs. Nat Cell Biol. (2022) 24:415–23. 10.1038/s41556-022-00880-5 35414016 PMC9035129

[32] Samir M Vidal RO Abdallah F Capece V Seehusen F Geffers R. Organ-specific small non-coding RNA responses in domestic (Sudani) ducks experimentally infected with highly pathogenic avian influenza virus (H5N1). RNA Biol. (2020) 17:112–24. 10.1080/15476286.2019.1669879 31538530 PMC6948974

[33] Kolliopoulou A Santos D Taning CN Wynant N Vanden Broeck J Smagghe G. pathway against viruses in insects. Wiley Interdisciplinary Reviews: RNA. (2019) 10:e1555. 10.1002/wrna.1555 31183996

